# Dynamic Subcellular Localization, Accumulation, and Interactions of Proteins From *Tomato Yellow Leaf Curl China Virus* and Its Associated Betasatellite

**DOI:** 10.3389/fpls.2020.00840

**Published:** 2020-06-16

**Authors:** Hao Li, Fangfang Li, Mingzhen Zhang, Pan Gong, Xueping Zhou

**Affiliations:** ^1^State Key Laboratory for Biology of Plant Diseases and Insect Pests, Institute of Plant Protection, Chinese Academy of Agricultural Sciences, Beijing, China; ^2^State Key Laboratory of Rice Biology, Institute of Biotechnology, Zhejiang University, Hangzhou, China

**Keywords:** tomato yellow leaf curl China virus, tomato yellow leaf curl China betasatellite, dynamic subcellular localization, protein accumulation, interaction assay

## Abstract

Geminiviruses contain the largest number of species of plant viruses, and cause devastating crop diseases worldwide. The development of resistance to these viruses will require a clear understanding of viral protein function and interactions. Tomato yellow leaf curl China virus (TYLCCNV) is a typical monopartite geminivirus, which is associated with a tomato yellow leaf curl China betasatellite (TYLCCNB) in the field; the complex infection of TYLCCNV/TYLCCNB leads to serious economic losses in solanaceous plants. The functions of each protein encoded by the TYLCCNV/TYLCCNB complex have not yet been examined in a targeted manner. Here, we show the dynamic subcellular localization and accumulation of six viral proteins encoded by TYLCCNV and the βC1 protein encoded by TYLCCNB in plants over time, and analyzed the effect of TYLCCNV or TYLCCNV/TYLCCNB infection on these parameters. The interaction among the seven viral proteins was also tested in this study: C2 acts as a central player in the viral protein interaction network, since it interacts with C3, C4, V2, and βC1. Self-interactions were also found for C1, C2, and V2. Together, the data presented here provide a template for investigating the function of viral proteins with or without viral infection over time, and points at C2 as a pivotal protein potentially playing a central role in the coordination of the viral life cycle.

## Introduction

Geminiviruses are a group of plant viruses with single-stranded circular DNA genomes that are encapsidated in twinned viral particles, transmitted by insect vectors and causing devastating crop diseases worldwide. Based on their genome organization, transmission insect vectors, and host range, geminiviruses are divided into nine genera^1^. The genus *Begomovirus* comprises by far the largest number of species, many of which cause devastating diseases in economically important crops. The genome of bipartite begomoviruses consists of two DNA molecules, referred to as DNA A and DNA B, while that of monopartite begomoviruses consists of one DNA molecule, similar to the DNA A. Many monopartite begomoviruses are associated with betasatellites, forming a disease complex in the field (Zhou, 2013).

Tomato yellow leaf curl China virus (TYLCCNV) is a typical monopartite geminivirus, which appears associated with a tomato yellow leaf curl China betasatellite (TYLCCNB) in the field (Cui et al., 2004). Tobacco or tomato plants infected by TYLCCNV/TYLCCNB display dwarfing, leaf curling, yellow mosaic patterns and stem distortion symptoms. However, infection by TYLCCNV alone in tobacco or tomato plants fails to induce any obvious symptoms and accumulate less viral DNA (Cui et al., 2004). The TYLCCNV genome contains six open reading frames (ORFs), two in the virion sense orientation (V1 and V2) and four in the complementary sense orientation (C1, C2, C3, and C4), while TYLCCNB encodes the βC1 protein in the complementary sense orientation. The functions of βC1 are well characterized (Zhou, 2013; Li et al., 2018a); for example, TYLCCNB βC1 suppresses transcriptional gene silencing (TGS) by inhibiting the activity of *S*-adenosylhomocysteine hydrolase (SAHH) to restrict the production of *S*-adenosyl methionine, an essential methyltransferase co-factor (Yang et al., 2011); TYLCCNB βC1 also inhibits post-transcriptional gene silencing (PTGS) by up-regulating the expression of an endogenous RNA silencing suppressor, *rgs-CaM*, which can not only reduce the transcription of *NbRDR6* (Li et al., 2014), but also interact with NbSGS3 to guide its autophagic degradation (Li et al., 2017).

Although the function of TYLCCNV-encoded proteins was not investigated in detail, potential roles have been assigned to their positional homologs in other geminivirus species. For example, C1 encodes a replication initiator protein (Rep), which is required for the replication of the viral DNA. C1 does not possess DNA polymerase activity, and heavily relies on the host DNA replication machinery for viral multiplication. Therefore, C1 can interact with host factors and recruit them to replicate the viral genome; these factors include retinoblastoma-related proteins (RBR), which negatively regulate cell cycle (Kong et al., 2000). C2 encodes a transcriptional activator protein (TrAP), which transactivates expression of the coat protein (CP) and movement protein (MP) genes (Sunter and Bisaro, 1997). Interestingly, beet curly top virus (BCTV) C2 has a novel function in the promotion of viral replication, probably by restoring the DNA replication competency of the infected cells and thus creating a favorable cell environment for viral spread. Therefore, C2 seems to have a broad impact on the replication of geminiviruses, and this mechanism might have important epidemiological implications (Trinks et al., 2005; Caracuel et al., 2012). C3 is a replication enhancer protein (REn), which promotes the accumulation of the virus. C4 is found to be a major symptom determinant (Lai et al., 2009). V1, as the viral coat protein (CP), is responsible for packing viral particles, and can also serve as the viral MP to mediate the shuttle of viral DNA/protein complex from the nucleus to the cytoplasm (Zhou et al., 2011). Besides these roles, geminiviral V1 is the protein recognized by the insect vector during virus acquisition, and hence determines vector specificity (Briddon et al., 1990). V2 is an RNA silencing suppressor (Glick et al., 2008; Zhang et al., 2012), and in some monopartite begomoviruses is involved in the movement of the virus and capable of inducing a hypersensitive response (HR) (Harrison and Robinson, 2002; Mubin et al., 2010; Matić et al., 2016).

In addition to performing essential functions to facilitate viral replication, transcription, movement, or spread, viral proteins can exert other roles. For example, viral proteins can counter host defenses including TGS, PTGS, HR, and protein degradation mechanisms. It has been found that C1, C2, C4, and V2 can suppress TGS, and C2, C4, and V2 can suppress PTGS. C1 reduces the expression of the plant maintenance DNA methyltransferases, METHYLTRANSFERASE 1 (MET1) and CHROMOMETHYLASE 3 (CMT3), in both locally and systemically infected tissues, in turn suppressing TGS (Rodríguez-Negrete et al., 2013). The tomato yellow leaf curl virus (TYLCV) V2 protein interacts with the host histone deacetylase 6 and inhibits methylation-mediated TGS in plants (Wang et al., 2018); it also suppresses PTGS by interacting and interfering with SGS3 (Glick et al., 2008). The tomato leaf curl Yunnan virus (TLCYnV) C4 protein can impair the HIR1-mediated cell death through promoting the degradation of HIR1 via physical interaction with this host protein (Mei et al., 2019).

A clear and dynamic description of the subcellular localization of viral proteins is important to understand the virus infection cycle. For example, in animal viruses, changes in the subcellular localization of some CPs may be related to the progression of the viral infection (Wistuba et al., 1997). TYLCV V1 is localized in the nucleolus and weakly in the nucleoplasm when expressed alone, while it is re-localized outside of the nucleolus and into discrete nuclear foci in the presence of TYLCV (Wang et al., 2017). Protein–protein interactions among viral proteins also play pivotal roles during viral infection. For example, it has been revealed that the potyvirus turnip mosaic virus (TuMV) P3N-PIPO is a plasmodesmata (PD)-localized protein that physically interacts with the virus-encoded cylindrical inclusion (CI) protein *in planta*; the CI and P3N-PIPO complex coordinates the formation of PD-associated structures that facilitate the intercellular movement of TuMV in infected plants (Wei et al., 2010).

In this study, we fused a YFP tag to each protein encoded by TYLCCNV/TYLCCNB and then transiently expressed them in *Nicotiana benthamiana* plants. Subcellular localization and protein accumulation were examined in real-time in the presence or absence of the viral infection complex. We found that the fluorescent signal and protein accumulation of C1, C2, V1, and βC1 decreased, while those of C3 and V2 increased, and those of C4 did not display obvious changes after 48 h post infiltration. The concomitant viral infection enhanced the fluorescent signal and protein accumulation of most viral proteins (C1, C2, C4, V1, V2, and βC1). The potential interaction among viral proteins was tested by yeast two-hybrid (Y2H) and bimolecular fluorescence complementation (BiFC) assays; we found that the C2 protein can serve as a network hub by interacting with itself and several other viral proteins. Furthermore, we also observed the self-interactions of C1 and V2. In summary, our work provides useful data for further studies investigating the role of each geminiviral protein in the context of the viral infection.

## Materials and Methods

### Plant Materials and Growth Conditions

Wild-type and transgenic *N. benthamiana* seedlings expressing RFP-H2B (Martin et al., 2009; Anderson et al., 2018) were potted in soil and placed in an insect-free greenhouse at 25°C and 60% relative humidity under a 16 h light/8 h dark photo-period.

### Plasmid Constructs

The infectious clone plasmids pBinPLUS-Y10-1.7A, including 1.7 copies of the TYLCCNV isolate Y10 genome in tandem (10A), and pBinPLUS-Y10-1.7A+1.0b, including 1.7 copies of the TYLCCNV genome and 2 copies of the TYLCCNB sequences (10Aβ) in tandem, are described previously (Zhou et al., 2003). The full-length sequence information of TYLCCNV (accession number: AJ319675) and TYLCCNB (accession number: AJ781300) can be found in GenBank. Gateway technology was used in this study to construct all vectors required for Y2H assays, subcellular localization experiments, and BiFC experiments. The genomic DNA regions of C1, C2, C3, C4, V1, V2, and βC1 were amplified from the plasmid of pBinPLUS-Y10-1.7A+1.0b by PCR using the KOD high-fidelity enzyme (Takara, Beijing, China). The PCR products were subcloned into the pDONR221 vector by BP Clonase^®^ (Invitrogen, Beijing, China) following the manufacturer’s protocol. The resulting pDONR221 constructs were verified by sequencing and linearized by *Mlu*I. The linearized fragments including the specific DNA sequences were transferred by recombination to the indicated binary destination vectors using LR Clonase II (Invitrogen, Beijing, China). To construct plant transient expression plasmids expressing recombinant proteins tagged with C-terminal YFP, C-terminal YFP-N (YN), or C-terminal YFP-C (YC), the linearized fragments were recombined into the binary destination vector pEarleygate101, 35S-YN gateway, or 35S-YC gateway (Earley et al., 2006; Lu et al., 2010), respectively. To construct plasmids for Y2H experiments, these linearized fragments were recombined to pGADT7 gateway or pGBKT7 gateway vectors (Lu et al., 2010). All primers used in this study can be found in Supplementary Table S1.

### Y2H Assays

Y2H assays were performed as described previously (Shen et al., 2011). Briefly, the pGADT7-DEST and pGBKT7-DEST recombinant constructs expressing the viral proteins were co-transformed into yeast cells (strain Y2H gold), and plated on selective medium lacking tryptophan and leucine (SD/-Trp/-Leu) used to select for co-transformants. The co-transformed yeast cells were further diluted in the selective medium (SD/-Trp/-Leu) and the high-stringency selective medium lacking tryptophan, leucine, histidine, and adenine (SD/-Trp/-Leu/-His/-Ade) to test the interactions. Pictures were taken at 4 days after dilution.

### Confocal Microscopy

The observation of subcellular localization of viral proteins and BiFC assays was conducted by confocal microscopy as described previously (Li et al., 2018b). The genes of interest were transiently expressed in the transgenic RFP-H2B *N. benthamiana* leaves, and 1–2 cm^2^ sections of the infiltrated leaves were excised and examined by confocal microscopy (Carl Zeiss LSM T-PMT, Germany) at 48 h post-infiltration (hpi), 72 and 96 hpi. YFP was excited at 518 nm, and the emitted light was captured at 565–585 nm. RFP was excited at 552 nm, and the emitted light was captured at 590–630 nm. Generally, at least 20 cells were examined for each experiment. The collected images were analyzed using the ZEN 2 (Carl Zeiss Microscope GmbH-2011) software.

### Western Blot Analysis

Total proteins were extracted from the infiltrated areas expressing the viral factors at 48, 72, and 96 hpi, using protein extraction buffer [50 mM Tris-HCl (pH 6.8), 9 M urea, 4.5% SDS and 7.5% β-mercaptoethanol], as described previously (Xiong et al., 2009). Proteins were separated by electrophoresis in 12.5% SDS-PAGE, transferred to PVDF membranes, and probed with mouse anti-GFP monoclonal antibodies at a 1:8000 (v/v) dilution (Roche Applied Science, Basel, Switzerland). The secondary antibody was a goat anti-mouse IgG conjugated with peroxidase at 1:10000 (v/v) dilution (Cell Signaling Technology, Boston, MA, United States). Detection was performed using a high-sig ECL western blotting substrate (Tanon, Shanghai, China) by a chemiluminescence detection system (Tianneng, Shanghai, China). The relative quantification of the target protein in each gel lane was calculated using the Image J software as previously described (Shimoji et al., 2006), and the accumulation of viral proteins when co-infiltrated with mock solution were set to 100.

### RT-qPCR Analysis

Total RNA was extracted from the infiltrated leaves using Trizol reagent (Invitrogen, Carlsbad, CA, United States) and 1 mg of RNA was retrotranscribed into cDNA using the PrimeScript^TM^ reagent kit with gDNA eraser (TaKaRa, Dalian, Japan) according to the manufacturer’s protocol. The RT-qPCR was performed in triplicates using a Roche Light Cycler 96 system (Roche Applied Science, Basel, Switzerland) with the following program: 30 s at 95°C, 45 cycles of 5 s at 95°C, 30 s at 58°C, and 10 s at 72°C. The specificity of primer pairs was verified by RT-qPCR dissociation curve. The relative expression level was calculated using the comparative Cq (2^–ΔΔCq^) method. *NbActin* was used as an internal standard. The information of the primers used in the RT-qPCR experiments is listed in Supplementary Table S1.

## Results

### Subcellular Localization of TYLCCNV/TYLCCNB-Encoded Viral Proteins

To gain insight into the functions of the proteins encoded by TYLCCNV/TYLCCNB, their subcellular localization was analyzed. Firstly, the 7 viral open reading frames (ORFs) were cloned into pDNOR221 vector by BP reaction (Invitrogen), followed by recombination and in-frame fusion to the coding sequence of yellow fluorescence protein (YFP) by LR reaction (Invitrogen). The recombinant proteins were expressed transiently in leaves of transgenic RFP-H2B *N. benthamiana* plants which stably expressed RFP-H2B in the nucleus as a nuclear marker (Martin et al., 2009; Anderson et al., 2018), and fluorescent signals were observed at 48, 72, and 96 hpi, by confocal microscopy.

**FIGURE 1:**
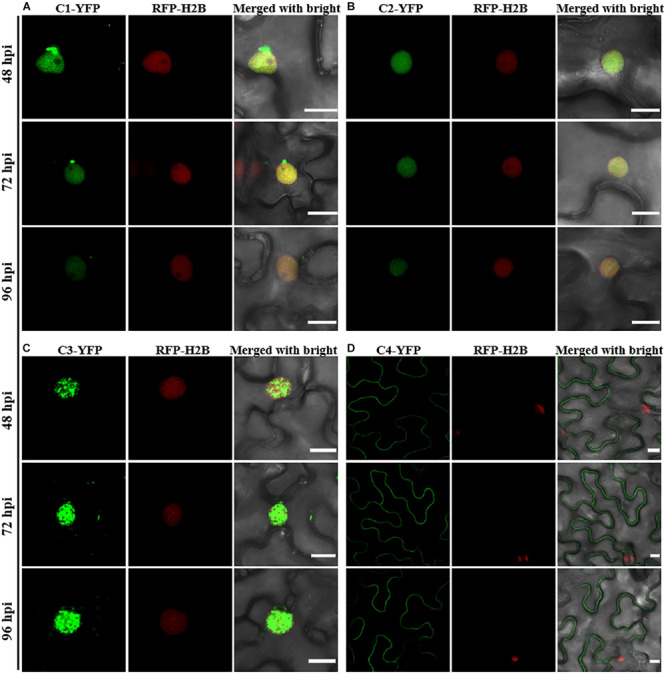
Confocal images of TYLCCNV C1, C2, C3, or C4 proteins fused with YFP. RFP-H2B transgenic *Nicotiana benthamiana* leaves were infiltrated with *Agrobacterium* cultures to express C1-YFP **(A)**, C2-YFP **(B)**, C3-YFP **(C)**, or C4-YFP **(D)**. Confocal images were taken at 48, 72, and 96 h post infiltration (hpi). YFP signal is shown in green. Nuclei of tobacco leaf epidermal cells are marked by RFP-H2B (red). This experiment was repeated three times independently, and more than 20 cells per sample were observed each time; representative results are shown. Scale bar: 10 μm.

**FIGURE 2:**
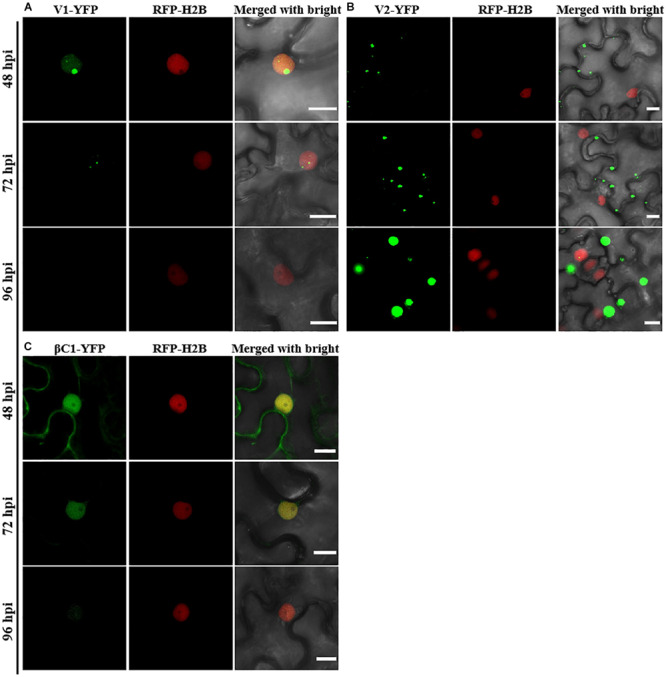
Confocal images of TYLCCNV V1, V2, or TYLCCNB βC1 fused with YFP. RFP-H2B transgenic *N. benthamiana* leaves were infiltrated with *Agrobacterium* cultures to express V1-YFP **(A)**, V2-YFP **(B)**, or βC1-YFP **(C)**. Confocal images were taken at 48, 72, and 96 hpi. YFP signal is shown in green. Nuclei of tobacco leaf epidermal cells are marked by RFP-H2B (red). This experiment was repeated three times independently, and more than 20 cells per sample were observed each time; representative results are shown. Scale bar: 10 μm.

Consistent with a previous study (Li et al., 2020), C1-YFP was mainly distributed in the nucleus at 48 hpi, but a few granules could be observed in the cytoplasm after 48 hpi (Figure 1A). C2-YFP was only present in the nucleus and did not change its localization over time, being uniformly distributed in this compartment (Figure 1B). Similar to C1-YFP, C3-YFP was mainly present in the nucleus, and C3-YFP-containing granules began to move out of the nucleus after 72 hpi. Interestingly, C3-YFP formed irregular and speckled granules in the nucleus, a distribution different to that of C1-YFP (Figure 1C). C4-YFP localized exclusively to the plasma membrane, as previously reported for other geminivirus C4 proteins (Mei et al., 2018), and this did not change in time (Figure 1D). V1-YFP was localized in the nucleoplasm and in the nucleolus at 48 hpi. The V1-YFP fluorescent signal decreased in the nucleoplasm, appearing in the nucleolus only, at 72 hpi. No fluorescence in V1-YFP-infiltrated leaves was observed at 96 hpi (Figure 2A). Different from the protein localization described above, V2-YFP formed some small granules in the cytoplasm, and the fluorescence of these granules became brighter after 48 hpi (Figure 2B). The βC1-YFP protein was distributed in both the nucleus and cytoplasm at 48 hpi (Figure 2C). It is worth noting that the fluorescence intensity of each YFP-fused protein changed differently over time (note: unless otherwise indicated, confocal images presented in the same figure panel were taken and processed with the same settings). For example, the fluorescent signal of C1-YFP, C2-YFP, V1-YFP, and βC1-YFP gradually decreased after 48 hpi (Figures 1, 2). However, the fluorescent signal of C3-YFP and V2-YFP gradually increased from 48 to 96 hpi (Figure 1). No obvious change in the fluorescence intensity of C4-YFP was observed from 48 to 96 hpi (Figure 1D).

### Dynamic Changes on the Accumulation of TYLCCNV/TYLCCNB-Encoded Proteins

**FIGURE 3:**
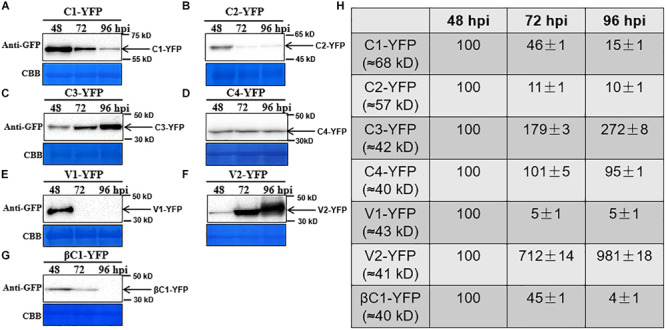
Dynamic changes in the accumulation of viral proteins. **(A–G)** *N. benthamiana* leaves were infiltrated with 0.5 ml of *Agrobacterium* cultures to express C1-YFP, C2-YFP, C3-YFP, C4-YFP, V1-YFP, V2-YFP, or βC1-YFP adjusted to an optical density at 600 nm (OD_600_) = 0.8. Total proteins were extracted from C1-YFP, C2-YFP, C3-YFP, C4-YFP, V1-YFP, V2-YFP, or βC1-YFP infiltrated zones at 48, 72, or 96 hpi. C1-YFP **(A)**, C2-YFP **(B)**, C3-YFP **(C)**, C4-YFP **(D)**, V1-YFP **(E)**, V2-YFP **(F)**, or βC1-YFP **(G)** at 48, 72, or 96 hpi were detected with an anti-GFP antibody. Arrows indicate the expected protein size. All immunoblotting assays in these figures were repeated at least three times, and each experimental sample was derived from three individual infiltrations. One representative blot is shown. Coomassie brilliant blue (CBB)-stained Rubisco large subunit was used as a loading control. **(H)** Accumulation of the specific proteins quantified from the western blot images by Image J, relative to the value of each protein at 48 hpi (set to 100).

To observe the dynamic changes in the accumulation of the TYLCCNV/TYLCCNB-encoded proteins, total proteins were extracted from C1-YFP, C2-YFP, C3-YFP, C4-YFP, V1-YFP, V2-YFP, or βC1-YFP-expressing leaves at 48, 72, and 96 hpi. Protein levels from 48 to 96 hpi are shown in Figure 3. The accumulation of C1-YFP, C2-YFP, V1-YFP, and βC1-YFP decreased after 48 hpi. The accumulation of C1-YFP, V1-YFP, and βC1-YFP was almost undetectable at 96 hpi (Figures 3A,B,E,G), while that of V1-YFP and βC1-YFP decreased significantly over time. The amount of C3-YFP and V2-YFP increased after 48 hpi (Figures 3C,F). No obvious change in the accumulation of C4-YFP was detected (Figure 3D). To evaluate the observed changes in more detail, we quantified the relative protein accumulation by Image J with the data at 72 and 96 hpi normalized to those at 48 hpi (set to 100) (Figure 3H). These data were consistent with the confocal observation. To further determine whether the decreased protein accumulation of viral proteins was due to decreased transcript accumulation. RT-qPCR analysis was carried out, and decreased RNA levels of *C1-YFP*, *C2-YFP*, *V1-YFP*, and β*C1-YFP* were observed from 48 to 96 hpi (Supplementary Figure S1), suggesting that RNA-mediated degradation was involved in lower accumulation of the protein.

### TYLCCNV/TYLCCNB Infection Enhances the Accumulation of Viral Proteins

**FIGURE 4:**
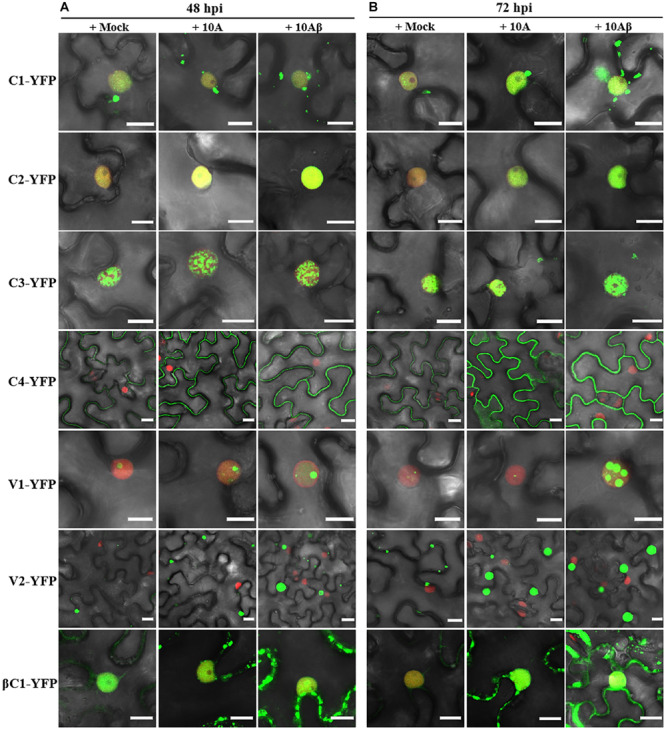
Confocal images of viral proteins fused with YFP in the presence or absence of TYLCCNV (10A), or TYLCCNV/TYLCCNB (10Aβ). **(A,B)** RFP-H2B *N. benthamiana* leaves were co-infiltrated with *Agrobacterium* cultures carrying the indicated constructs, and then examined by confocal microscopy at 48 hpi **(A)** and 72 hpi **(B)**. All *Agrobacterium* cultures were adjusted to an optical density at 600 nm (OD600) = 0.8 and 0.5 ml of *Agrobacterium* cultures were infiltrated into leaves. These experiments were repeated three times by three independent infiltrations, and more than 20 cells per sample were observed in each replicate; representative results are shown. Scale bar: 10 μm.

**FIGURE 5:**
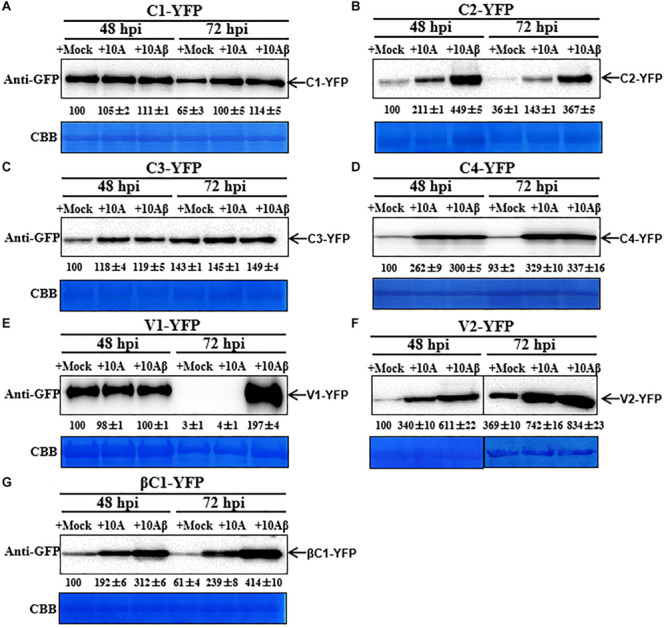
Dynamic changes in the accumulation of viral proteins in the presence or absence of TYLCCNV (10A) or TYLCCNV/TYLCCNB (10Aβ). Wild type *N. benthamiana* leaves were infiltrated with *Agrobacterium* cultures to express C1-YFP, C2-YFP, C3-YFP, C4-YFP, V1-YFP, V2-YFP, or βC1-YFP together with those harboring an empty vector (Mock), or TYLCCNV or TYLCCNV/TYLCCNB infectious clones. **(A–G)** C1-YFP, C2-YFP, C3-YFP, C4-YFP, V1-YFP V2-YFP, or βC1-YFP proteins detected by western blot. Total proteins were extracted from infiltrated leaves as indicated at 48 and 72 hpi, and detected with an anti-GFP antibody. The sample volume was 20 μl **(A–E)** or 10 μl **(F,G)**. All immunoblotting assays in these figures were repeated at least three times, and each experimental sample derived from three individual infiltrations. One representative blot is shown. CBB-stained Rubisco large subunit was used as a loading control. The values of C1-YFP, C2-YFP, C3-YFP, C4-YFP, V1-YFP, V2-YFP, or βC1-YFP at 48 hpi were quantified by Image J and set to 100 for normalization.

In order to better determine the localization and expression of each protein in the context of the virus infection, *Agrobacterium* cultures that express the YFP-fused viral proteins were co-infiltrated with *Agrobacterium* cultures carrying an empty vector (Mock), or TYLCCNV (10A), or TYLCCNV/TYLCCNB (10Aβ) infectious clones into RFP-H2B transgenic *N. benthamiana* leaves and infiltrated leaves were examined by confocal microscope at 48 and 72 hpi. Viral intercellular movement may take place after 72 hpi, with replicating viruses moving onto neighboring areas or systemic leaves from the infiltrated zones. Therefore, we chose 48 and 72 hpi, rather than 96 hpi, to analyze the effects of virus infection on the expression and subcellular location of TYLCCNV/TYLCCNB-encoded proteins in the infiltrated leaves. Co-infiltration with TYLCCNV or TYLCCNV/TYLCCNB had no obvious effect on the localization or protein accumulation of C1-YFP at 48 hpi compared to that with Mock (Figures 4A, 5A), but enhanced C1-YFP fluorescence and increased protein accumulation were observed at 72 hpi (Figures 4A, 5A). Increased fluorescent signal and protein accumulation of C2-YFP were observed in the presence of TYLCCNV or TYLCCNV/TYLCCNB at 48 and 72 hpi (Figures 4A,B, 5B). Similarly, co-expression with TYLCCNV or TYLCCNV/TYLCCNB increased the fluorescent signal and protein accumulation of C3-YFP, but to a lower extent (Figures 4A,B, 5B,C). It was also obvious that the virus infection enhanced the fluorescence intensity and increased the protein accumulation of C4-YFP at 48 and 72 hpi (Figure 5D). There was no significant difference in the subcellular localization and protein accumulation of V1-YFP in the presence or absence of TYLCCNV at 48 hpi (Figures 4A,B). However, TYLCCNV/TYLCCNB, but not TYLCCNV alone, at 72 hpi could change the subcellular localization and increase the protein accumulation of V1-YFP, which formed larger and more abundant granules in the nucleus, at 72 hpi. This result indicates that the enhanced fluorescence and increased protein level of V1-YFP at 72 hpi is dependent on TYLCCNB (Figure 5E). The presence of TYLCCNV or TYLCCNV/TYLCCNB increased the fluorescence and protein accumulation of V2-YFP and βC1-YFP at 48 and 72 hpi (Figures 4A,B, 5F,G). Taken together, these data indicate that the accumulation of all viral proteins is enhanced during the viral infection, especially in the presence of TYLCCNB.

### Interactions Among TYLCCNV/TYLCCNB-Encoded Proteins

**FIGURE 6:**
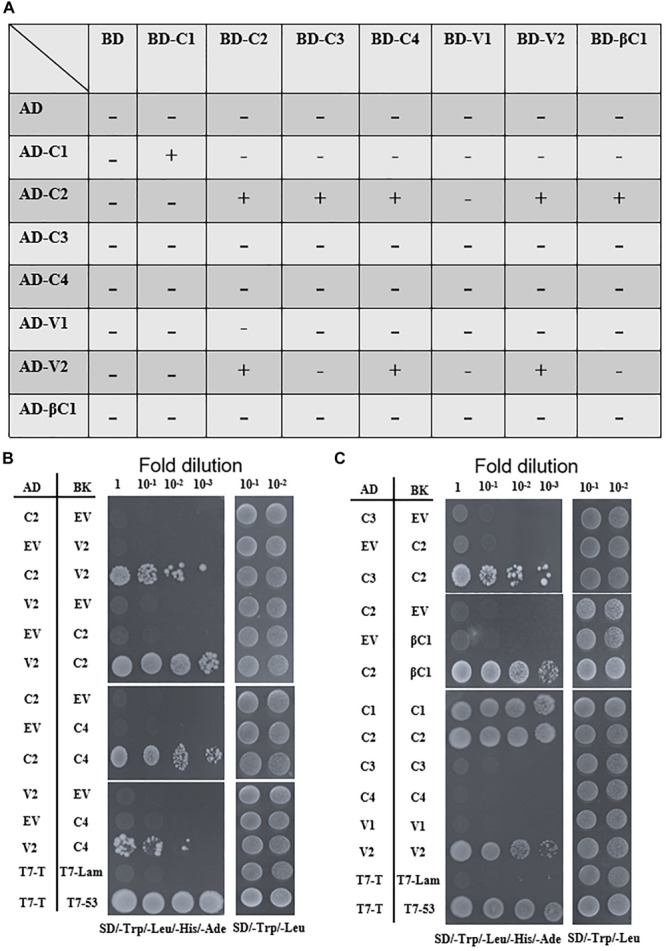
Yeast two-hybrid assays among TYLCCNV/TYLCCNB-encoded proteins. **(A)** Summary of Y2H assay results. “–” means no positive interaction was detected; “+” means a positive interaction was detected. The Y2H assays summarized in this table were repeated at least three times by each independent transformation. **(B)** Interactions between C2 and V2, C2 and C4, and V2 and C4 by Y2H. **(C)** Interactions between C2 and C3, C2 and βC1, and self-interactions of C1, C2, and V2 by Y2H. The Y2H Gold yeast strain cells co-transformed with the indicated plasmids were plated on synthetic dextrose (SD)/-Trp, -Leu, -His, -Ade medium to identify protein interactions. Photographs were taken at 4 days post-transformation. Yeast cells co-transformed with pGADT7-T and pGBKT7-Lam were used as negative controls, and with pGADT7-T and pGBKT7-53 were used as positive controls **(B,C)**.

To screen for possible interactions among the seven viral proteins encoded by TYLCCNV/TYLCCNB, yeast two-hybrid (Y2H) assays were conducted. All these viral proteins were fused with the GAL4 transcription activation domain (AD) and the GAL4 DNA binding domain (BD), respectively. As summarized in Figures 6A,B, interactions among TYLCCNV/TYLCCNB-encoded proteins were identified. The C2 protein acted as an interaction hub, since it can interact with most proteins including C3, C4, V2, and βC1 (Figures 6B,C). In addition, the interaction between C4 and V2, and self-interactions of C1, C2, and V2 were also found (Figures 6B,C). Unexpectedly, we did not detect interactions between the V1 (CP) with any other viral protein, or with itself, although the self-interaction of TYLCV V1 was previously reported (Hallan and Gafni, 2001).

**FIGURE 7:**
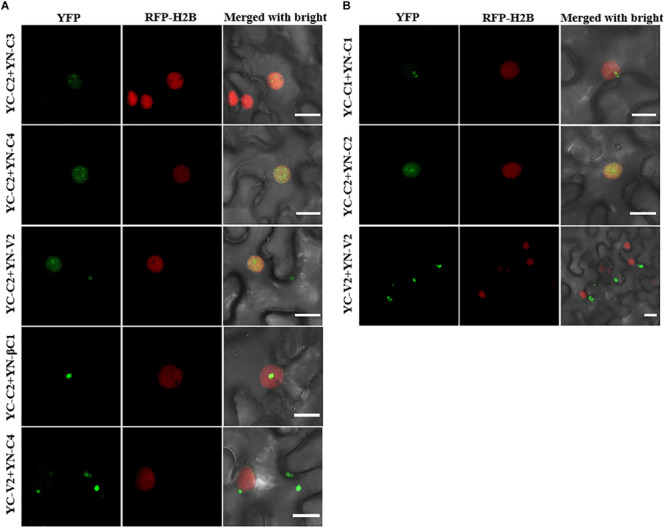
BiFC assays to confirm the interactions among TYLCCNV/TYLCCNB-encoded proteins. **(A)** Confocal visualization of the interaction between C2 and C3, C2 and C4, C2 and V2, C2 and βC1, and C4 and V2 in RFP-H2B transgenic *N. benthamiana* leaves. The fused proteins indicated above were transiently expressed in RFP-H2B transgenic *N. benthamiana* leaves. Images were taken at 48 hpi. Yellow fluorescence (in green) was found in the leaf cells co-expressing C2 + C3, C2 + C4, C2 + V2, C2 + βC1, and C4 + V2. Nuclei of tobacco leaf epidermal cells are indicated by RFP-H2B (red). Bars, 10 μm. **(B)** Confocal visualization of the self-interaction of C1, C2, and V2. C1, C2, and V2 were fused with YN or YC, respectively. Yellow fluorescence (in green) was found upon pairwise expression of YN-C1 + YC – C1, YN – C2 + YC – C2, and YN – V2 + YC – V2 at 48 hpi. Bars, 10 μm. These experiments were repeated three times by three independent infiltrations; one representative result is shown **(A,B)**.

All the interactions identified by Y2H were further confirmed by bimolecular fluorescence complementation (BiFC) in transgenic *N. benthamiana* plants expressing RFP-H2B as a nuclear marker. The C2–C3, C2–C4, and C2–βC1 interactions, as well as the C1 and C2 self-interactions, were detected in the nucleus (Figures 7A,B). The interaction between C2 and V2 was localized in the nucleus and in the cytoplasm (Figure 7A). The interaction between C4 and V2, and the V2 self-interaction were observed in the cytoplasm (Figures 7A,B). As a negative control, no YFP fluorescence was observed when the movement protein P3N-PIPO from TuMV (Wei et al., 2015) was co-expressed with C1, C2, C3, C4, V2, or βC1 (Supplementary Figure S2).

## Discussion

Although the subcellular localization of many geminivirus proteins has been analyzed, the subcellular localization of TYLCCNV/TYLCCNB-encoded proteins has not been reported yet. In this study, the subcellular localization of all TYLCCNV/TYLCCNB-encoded proteins and the effects of the viral infection on the subcellular localization and accumulation of each protein were investigated. C1, as a replication initiation protein (Rep) involved in the replication of the geminivirus genome in the nucleus, was initially localized in this subcellular compartment, and then partially localized outside of the nucleus with decreasing fluorescence intensity over time (Figure 1). We found that both in the protein level and RNA level of TYLCCNV C1-YFP decreased from 48 to 96 hpi (Figure 3 and Supplementary Figure S1), suggesting that the expression of C1-YFP is susceptible to both RNA and protein-mediated degradation. Similarly, C2-YFP was also localized in the nucleus with decreasing fluorescence intensity, RNA accumulation and protein accumulation after 48 hpi (Figures 1, 3). In contrast, C3-YFP formed speckled granules in the nucleus with increasing fluorescence intensity and protein accumulation, showing that C3 is probably resistant to host RNA and protein-mediated degradation mechanisms. C4-YFP was localized at the plasma membrane, and no obvious change was observed in the C4-YFP subcellular localization or C4-YFP protein accumulation over time (Figures 2, 3). Decreased fluorescent signal, protein accumulation and RNA levels of V1-YFP and βC1-YFP were also observed (Figures 2, 3 and Supplementary Figure S1). Based on the above results, we speculate that transiently expressed viral proteins can be targets of host RNA and protein degradation systems. Three major RNA degradation pathways including RNA silencing, RNA decay, and RNA quality control can target exogenous RNAs in plants, and mediate degradation of target transcripts (Li and Wang, 2019). The ubiquitin-proteasome system (UPS) and autophagy are reported to be involved in the degradation of many viral protein in plants (Shen et al., 2016; Cheng and Wang, 2017; Haxim et al., 2017; Yang et al., 2018; Li et al., 2020). For example, autophagy was involved in the degradation of several geminivirus C1 proteins, including C1 of TLCYnV, TYLCV, tobacco curly shoot virus (TbCSV), and mungbean yellow mosaic India virus (MYMIV), which directly interacted with the autophagy protein ATG8h (Li et al., 2020). The geminivirus βC1 can be the target of both the UPS and autophagy, which leads to its degradation (Shen et al., 2016; Haxim et al., 2017). In addition, TYLCV infection in whiteflies activated autophagy to lead to the degradation of the V1 protein and viral genomic DNA (Wang et al., 2016). However, an increased number of fluorescent aggregates and higher protein accumulation of V2-YFP were observed in our experiments (Figures 2, 3), suggesting that V2 can suppress host RNA or protein degradation mechanisms. Indeed, TYLCCNV and TYLCV V2 acts as a strong RNA silencing suppressor (Glick et al., 2008; Zhang et al., 2012).

The observation of the subcellular localization of viral proteins in the context of the viral infection is important to understand their biological roles. In this study, we noticed that the fluorescent signals of almost all viral proteins can be enhanced when TYLCCNV alone or TYLCCNV/TYLCCNB are present, especially the fluorescent intensity of V1-YFP and βC1-YFP (Figures 4, 5), which were consistent with their protein accumulation. Viral proteins encoded in TYLCCNV or TYLCCNB such as V2 and βC1, are able to suppress RNA silencing (Cui et al., 2005; Zhang et al., 2012), so it is reasonable that co-infection of TYLCCNV or TYLCCNV/TYLCCNB enhances the fluorescence intensity and protein expression. It is worth noting that the V1-YFP protein levels were particularly sensitive to TYLCCNV/TYLCCNB infection. At 72 hpi, different from other viral proteins, TYLCCNV alone could not block the sharp decrease of the V1-YFP protein accumulation, while TYLCCNV/TYLCCNB could increase it to a higher level compared to that at 48 hpi (Figure 5E). These results indicate that TYLCCNV/TYLCCNB is required for the accumulation of the V1-YFP protein. In addition, V1-YFP was localized in the nucleoplasm and nucleolus, while TYLCCNV/TYLCCNB infection caused the appearance of more bright granules in the nucleus (Figure 4). Geminivirus infection changed the subnuclear localization of V1, which has also been reported previously (Wang et al., 2017), suggesting the conserved functions of different geminiviruses. Except for V1, there was no obvious difference in the subcellular localization observed for TYLCCNV/TYLCCNB-encoded proteins in the presence of TYLCCNV alone or TYLCCNV/TYLCCNB, indicating that the unique localization of viral proteins contributes to the viral infection.

Protein–protein interactions during the viral infection are important ways to link different viral proteins together to cooperatively participate in the viral life cycle. We found that several interactions among different viral proteins exist, and C2 serves as an interaction hub, associating with most viral proteins (Figures 6, 7). The geminivirus transcriptional activator C2 protein is a multifunctional protein, which can bind DNA, suppress TGS and PTGS, and also act as a pathogenicity factor (Noris et al., 1996; van et al., 2002; Fondong, 2013). For example, C2 positional homologs of some geminiviruses interact with and inactivate adenosine kinase (ADK) which is required for efficient production of *S*-adenosyl methionine, an essential methyltransferase cofactor. Therefore, C2 homologs can suppress TGS and cause a genome-wide reduction in cytosine methylation (Buchmann et al., 2009). C2 encoded by beet severe curly top virus (BSCTV) interacts with *S*-adenosyl-methionine decarboxylase 1 (SAMDC1) and interferes with the host defense mechanism of DNA methylation-mediated gene silencing through attenuating its 26S proteasome-mediated degradation of SAMDC1 (Zhang et al., 2011). Therefore, the interaction between C2 and C3 probably facilitates the replication of the viral genome and the transcription of viral RNAs by suppressing host TGS and PTGS responses. Geminivirus C4, V2, or βC1 can repress many host defenses with different mechanisms (Zhang et al., 2012; Li et al., 2018a; Wang et al., 2018; Fondong, 2019), so the interactions between C2 and C4, C2 and V2, or C2 and βC1 might enhance viral pathogenicity by forming strong counter-defense complexes to suppress host defense responses. The interaction between V2 and C4 was observed as bright granules localized in the cytoplasm (Figure 5A). V2 has been reported as involved in viral movement, and is also able to suppress TGS and PTGS. For example, cotton leaf curl Multan virus (CLCuMuV) V2 protein inhibits TGS by interacting with AGO4 (Wang et al., 2019); TYLCV V2 can achieve PTGS inhibition by interacting with the host SGS3 (Glick et al., 2008), and interacts with *N. benthamiana* histone deacetylase 6 to inhibit methylation-mediated TGS (Wang et al., 2018). C4 has been reported as a movement protein (Jupin et al., 1994; Rojas et al., 2001), so the interaction between C4 and V2 may participate in the movement of the virus and counter host defense responses. Based on the subcellular localization of V2 and C4, we speculate that V2 probably transports the encapsulated virus particles to the cell membrane, where C4 transports them to the neighboring cells.

Self-interactions of TYLCCNV C1, C2, and V2 were also found (Figures 6, 7). It has been shown that the self-interaction of C1 encoded by abutilon mosaic virus (AMV) is involved in initiating the replication in the nucleus (Krenz et al., 2011). Geminivirus C1 is a multifunctional protein with site-specific nicking and ligase activity, helicase activity, site-specific DNA binding activity, and ATPase activity, and it can recruit several host proteins to usurp the host replication machinery for viral proliferation (Hanley-Bowdoin et al., 2013). Therefore, the self-interaction of TYLCCNV C1 is probably involved in its multifunctional activities, which are necessary for the establishment of functional virus replication complexes. The TYLCCNV C2–C2 interaction complexes accumulated primarily in the nucleus, which was consistent with the previous observation that the self-interaction of C2 positional homologs correlates with their nuclear localization and efficient activation of transcription, whereas their monomers can suppress local silencing by interacting with ADK in the cytoplasm (Yang et al., 2007). The self-interaction of TYLCCNV V2 was also observed in this study (Figures 6, 7), similar to what has been observed for TYLCV V2. A single amino acid in V2 encoded by TYLCV is responsible for its self-interaction, aggregates formation, and pathogenicity (Zhao et al., 2018). The self-interaction of TYLCV CP (V1) was found in a previous report, which identified the N-terminal region of CP as necessary (Hallan and Gafni, 2001). However, no self-interaction of TYLCCNV V1 was found in this study. One possible explanation is sequence differences between these viral proteins, since TYLCCNV V1 shares 76.7% amino acid identity to TYLCV V1 (Supplementary Figure S3).

In brief, we have determined the dynamic localization and protein accumulation of TYLCCNV/TYLCCNB-encoded proteins over time with or without the viral infection, and explored the potential roles of each protein in virus replication and infection based on their interactions. Therefore, this study provides solid and useful information for further works on the biological significance of the viral proteins during the viral infection.

## Data Availability Statement

The datasets generated for this study can be found in the TYLCCNV (accession number: AJ319675) and TYLCCNB (accession number: AJ781300) can be found in GenBank.

## Author Contributions

HL, FL, MZ, and PG performed all experiments. FL, HL, and XZ contributed to experimental design and interpretation. FL and XZ conceived the project. HL, FL, and XZ wrote the manuscript with contributions from all authors.

## Conflict of Interest

The authors declare that the research was conducted in the absence of any commercial or financial relationships that could be construed as a potential conflict of interest.

## Footnotes

1 https://talk.ictvonline.org/taxonomy/

## Supplementary Material

FIGURE S1 Dynamic changes in the transcripts of viral genes. RT-qPCR analysis of the YFP RNA fused with C1, C2, V1, and βC1. The values of C1-YFP, C2-YFP, V1-YFP, and βC1-YFP at 48 hpi were normalized against *NbActin* transcripts in the same sample. Three independent experiments, each consisting of three biological replicates, were carried out. Values from one representative result were used to plot a histogram. Statistical analysis was performed using GraphPad Prism 6 software followed by ANOVA using Student’s *t*-test (two-sided, **P* < 0.05, ***P* < 0.01, ****P* < 0.001).

FIGURE S2 Interaction assays of P3N-PIPO and C1, C2, C3, C4, V2, or βC1. BiFC assays in RFP-H2B transgenic *N. benthamiana* leaves expressing P3N-PIPO and C1, C2, C3, C4, V2, or βC1 at 48 h post infiltration (hpi). Bars = 20 μm. No YFP fluorescence was detected.

FIGURE S3 Amino acid sequence alignment of TYLCV-V1 and TYLCCNV-V1 was performed in MegAlign (Lasergene) and sequence distances were conducted in MegAlign using the Clustal V Method.

TABLE S1 Primers used in plasmid construction and other experiments in this study.
