# Supplementary material for: Dynamic Subcellular Localization, Accumulation, and Interactions of Proteins From Tomato Yellow Leaf Curl China Virus and Its Associated Betasatellite
Source: Front Plant Sci. 2020 Jun 16;11:840. doi: 10.3389/fpls.2020.00840 (PMC7308551; doi:10.3389/fpls.2020.00840)
Supplement: FIGURE S1 — Dynamic changes in the transcripts of viral genes. RT-qPCR analysis of the YFP RNA fused with C1, C2, V1, and βC1. The values of C1-YFP, C2-YFP, V1-YFP, and βC1-YFP at 48 hpi were normalized against NbActin transcripts in the same sample. Three independent experiments, each consisting of three biological replicates, were carried out. Values from one representative result were used to plot a histogram. Statistical analysis was performed using GraphPad Prism 6 software followed by ANOVA using Student’s t-test (two-sided, *P < 0.05, **P < 0.01, ***P < 0.001). [file Table_1.docx]

| Prime Name | Sequence |
| --- | --- |
| 221-C1-F | GGGGACAAGTTTGTACAAAAAAGCAGGCTTCATGCCTCCTCCTAATAAATT |
| 221-C1-R | GGGGACCACTTTGTACAAGAAAGCTGGGTCACCCGACGTCGTCTGGTTGT |
| 221-C2-F | GGGGACAAGTTTGTACAAAAAAGCAGGCTTCATGCGATCTTCGTCACCCTC |
| 221-C2-R | GGGGACCACTTTGTACAAGAAAGCTGGGTCAATACTCTTAAGAAACGCAA |
| 221-C3-F | GGGGACAAGTTTGTACAAAAAAGCAGGCTTCATGGATTCACGCACCGGGGA |
| 221-C3-R | GGGGACCACTTTGTACAAGAAAGCTGGGTCATAAATATTAAATTTTATAT |
| 221-C4-F | GGGGACAAGTTTGTACAAAAAAGCAGGCTTCATGGGACTCCTCACCTGCAT |
| 221-C4-R | GGGGACCACTTTGTACAAGAAAGCTGGGTCATATATTGAGGGCCGAAGCT |
| 221-V1-F | GGGGACAAGTTTGTACAAAAAAGCAGGCTTCATGTCGAAGCGTCCCGCAGA |
| 221-V1-R | GGGGACCACTTTGTACAAGAAAGCTGGGTCATTCAACTGAGAATCATAAA |
| 221-V2-F | GGGGACAAGTTTGTACAAAAAAGCAGGCTTCATGTGGGATCCTCTGCTCAA |
| 221-V2-R | GGGGACCACTTTGTACAAGAAAGCTGGGTCGGGCTTCTGTACATCCGATA |
| 221-βC1-F | GGGGACAAGTTTGTACAAAAAAGCAGGCTTCATGACTATCAAATACAACAA |
| 221-βC1-R | GGGGACCACTTTGTACAAGAAAGCTGGGTCTACATCTGAATTTGTAAATACAT |
| M13F | GTTGTAAAACGACGGCCAG |
| M13R | CAGGAAACAGCTATGAC |
| 35S-F | CGCAAGACCCTTCCTCTATATAAGGAA |
| AD/BD-T7 | TAATACGACTCACTATAGGG |
| C1-R | ACGTCGTCTGGTTGTTTTCT |
| C2-R | ACGCAAGGTCTGAGGATGTA |
| C3-R | TGTGCCTTCTAGTACATTGT |
| C4-R | ATATTGAGGGCCGAAGCTTT |
| V1-R | AGTCGCATATACAGGATTAC |
| V2-R | ACATCCGATACATCATGGGC |
| βC1-R | GTTGAAGTCGAATGGTGGGA |
| q-Nbactin-F | AAAGACCAGCTCATCCGTGGAGAA |
| q-Nbactin-R | TGTGGTTTCATGAATGCCAGCAGC |
| q-YFP-F | CATGAAGCAGCACGACTTCT |
| q-YFP-R | CTGCTTGTCGGCCATGATATAG |
